# Deep learning models for predicting the survival of patients with medulloblastoma based on a surveillance, epidemiology, and end results analysis

**DOI:** 10.1038/s41598-024-65367-9

**Published:** 2024-06-24

**Authors:** Meng Sun, Jikui Sun, Meng Li

**Affiliations:** Department of Neurosurgery, The First Affiliated Hospital of Shandong First Medical University, Jinan, 250014 Shandong China

**Keywords:** DeepSurv, Medulloblastoma, Neural network, Survival prediction, SEER, Cancer models, CNS cancer

## Abstract

Medulloblastoma is a malignant neuroepithelial tumor of the central nervous system. Accurate prediction of prognosis is essential for therapeutic decisions in medulloblastoma patients. We analyzed data from 2,322 medulloblastoma patients using the SEER database and randomly divided the dataset into training and testing datasets in a 7:3 ratio. We chose three models to build, one based on neural networks (DeepSurv), one based on ensemble learning that Random Survival Forest (RSF), and a typical Cox Proportional-hazards (CoxPH) model. The DeepSurv model outperformed the RSF and classic CoxPH models with C-indexes of 0.751 and 0.763 for the training and test datasets. Additionally, the DeepSurv model showed better accuracy in predicting 1-, 3-, and 5-year survival rates (AUC: 0.767–0.793). Therefore, our prediction model based on deep learning algorithms can more accurately predict the survival rate and survival period of medulloblastoma compared to other models.

## Introduction

Medulloblastoma is an embryonal tumor that arises from the cerebellum and has the potential to spread throughout the nervous system. It is the most common type of paediatric embryonal tumor, with an incidence ranging from 5 to 11 cases per 1 million individuals^1,2^. According to current international consensus, there are four subgroups of medulloblastoma: Wingless (WNT), Sonic Hedgehog (SHH), group 3 (G3), and group 4 (G4)^3^. Multimodal therapy, which includes surgery, external beam irradiation, and/or cytotoxic chemotherapy, can result in survival rates ranging from 50 to 80% based on clinical staging^4^. Certain prognostic features, such as age at diagnosis, extent of resection, histological subtype, and molecular subgroup classification, have been found to affect survival predictions in individual patients.

Previous studies have used the Cox proportional-hazards model (CoxPH) to evaluate the survival rate of medulloblastoma patients^5–7^. This model incorporates survival outcomes and time as target variables, allowing for the simultaneous analysis of multiple factors' impact on survival time. It is extensively used for predicting outcome events when the survival distribution of the analyzed data is unknown^8^. A nomogram is a commonly used method for quantifying and combining important clinical characteristics of patients to calculate the probabilities of outcome events based on the CoxPH model^9^. However, the model assumes that each predictor variable has the same effect throughout the follow-up time, which ignores variations in their impact on individual patients at different times. Therefore, a new method is required to improve the accuracy of predicting the survival rate of cancer patients.

In recent years, computer and information technology have shown revolutionary potential for artificial intelligence (AI) in the healthcare industry^10–12^. Machine learning models have stronger nonlinear modeling capabilities compared to traditional linear models and can better capture complex relationships among clinical variables. The analysis of these models can provide accurate personalized survival predictions and decision-making support for treatment strategies to improve patient survival rates^13,14^. Deep learning is a subfield of machine learning that involves discovering the distributed features of sample data by learning the underlying laws and levels of representation^15,16^. Neural networks are at the heart of deep learning algorithms and consist of input, hidden and output layers that can be used to solve complex, multi-factor and non-linear problems. Deep learning-based models have become highly effective predictors of clinical outcomes across various disease domains due to the continuous advancements in deep learning research techniques and the abundance of biomedical big data. Jiang et al.^17^ demonstrated the use of an artificial neural network model to predict the survival rate of patients diagnosed with pancreatic neuroendocrine neoplasms, by leveraging clinical information. Katzman et al.^18^ integrated deep learning with a multilayer neural network architecture, known as the DeepSurv model, resulting in a personalized treatment recommendation system that showed remarkable performance.

To our knowledge, there is a lack of research combining deep learning techniques with the study of medulloblastoma. Therefore, this study aimed to fill this research gap by utilizing data obtained from the Surveillance, Epidemiology, and End Results (SEER) database, which contains information on patients diagnosed with medulloblastoma in the United States. And then the DeepSurv model was used to evaluate their survival rates.

## Method

### Data source and patient selection

The data of this retrospective cohort study were obtained from the SEER database, which encompasses information from 18 cancer registries representing approximately 28% of the entire US population^19^. This database offers extensive and detailed patient data, including demographic characteristics, tumor-related information, cause of death, and survival duration. The SEER*Stat software (version 8.3.6) was used to identify patients with medulloblastoma. The dataset covering the years 2000 to 2019 in the United States was accessed.

**Figure 1:**
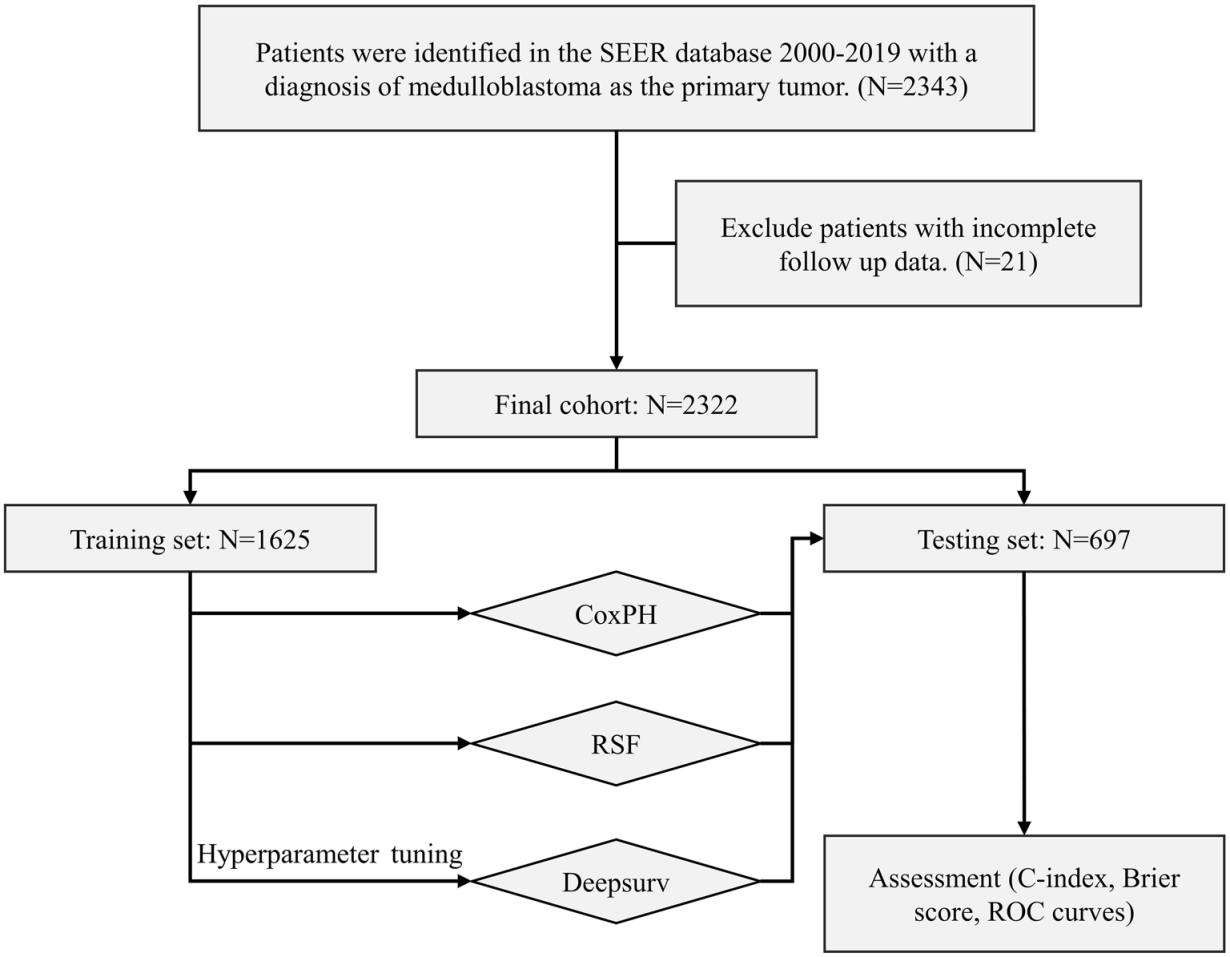
Study profile and analysis pipeline. Patients with a diagnosis of medulloblastoma as primary tumor in the SEER database 2000–2019 with complete follow-up data. The entire dataset was divided 7:3 into training (n = 1,625) and test (n = 697) sets. The CoxPH and RSF models were constructed directly from the training set data. When constructing the Deepsurv model, we used grid search and fivefold cross-validation for hyperparameter tuning on the training dataset. Finally, the performance of the models was evaluated in the testing set (n = 697) using several metrics.

The patients included in the study had to meet the following criteria: (1) a confirmed pathological diagnosis of medulloblastoma; (2) identification of medulloblastoma cases based on the third edition of the International Classification of Diseases for Oncology (ICD-O3) using specific ICD-O-3 codes for histopathology, including 9,470/3 for medulloblastoma, NOS; 9,471/3 for desmoplastic nodular medulloblastoma; and 9,474/3 for large cell medulloblastoma. Furthermore, patients were required to have a known survival status and time. Afterwards, they were randomly divided into a training group and a testing group at a 7:3 ratio. A flowchart in Fig. 1 illustrates the process of patient selection.

### Variable’s definitions

**Figure 2:**
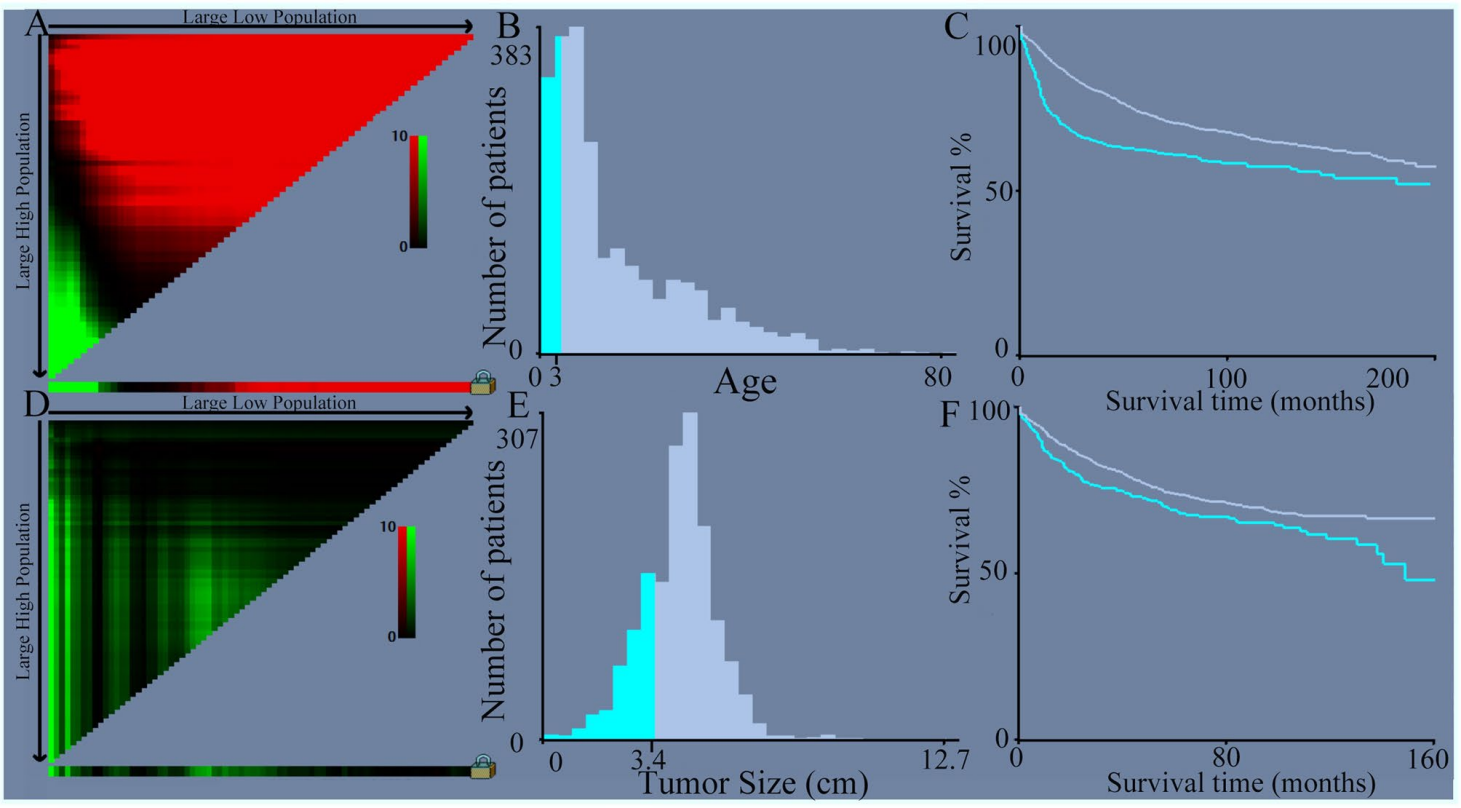
The X-tile analysis was conducted to determine the best cutoff points for the variables of age and tumor size. (**A**) X-tile plot of training sets in age. (**B**) The cutoff point highlighted using a histogram of the entire cohort. (**C**) Kaplan–Meier plot showing the distinct prognosis determined by the cutoff point. (**D**) X-tile plot of training sets in tumor size. (**E**) The cutoff point highlighted using a histogram. (**F**) Kaplan–Meier plot showing the prognosis determined by the cutoff point. The low subset is depicted in gray, while the high subset is shown in blue.

Several parameters were collected from the samples, including age at diagnosis, sex, race, histological type, tumor size, surgery, chemotherapy, radiation therapy, and survival time. To evaluate the prognostic value of age and tumor size in patients with medulloblastoma objectively, the patients were categorized into two groups based on the optimal cutoff values obtained using the X-tile software (https://x-tile.software.informer.com, Yale School of Medicine, New Haven, CT, United States). Age cutoff values of ≤ 3 years and > 3 years, and tumor size cutoff values of ≤ 3.4 cm, > 3.4 cm, and/or unknown were utilized. For detailed visual representations, please refer to Fig. 2.

### Model development

This study selected three models for training: DeepSurv, RSF, and CoxPH. DeepSurv is a deep feedforward neural network used to predict patients’ survival time or survival probability. It employs a multi-layer neural network to capture the complex nonlinear relationship between patients’ survival probability and input features. This study utilized deep-learning calculations based on the DeepSurv calculation method described by Katzman et al.^18^ to predict the survival outcome of patients diagnosed with medulloblastoma. The term RSF refers to Random Survival Forests, which is a survival analysis method based on random forests. When constructing a random survival forest, subsets of samples and features are randomly selected, and multiple decision trees are built using these subsets^20^. Each decision tree splits the samples based on features in the nodes and determines the optimal splitting based on the evaluation of survival time differences. The predictions from multiple decision trees in the random survival forests are combined to obtain the final survival prediction. The CoxPH is a semi-parametric regression model used to analyse survival data and estimate the risk of event occurrence. The Cox proportional-hazards model is used to compare the relative risks of events between different groups and study the impact of various factors on event occurrence. The model functions by modeling the relationship between time and event occurrence as a function of hazard ratios.

We performed hyperparameter tuning in the Deepsurv model using grid search and fivefold cross-validation on the training dataset, selecting the parameter with the highest average C-index in the cross-validation as the optimal parameter.

For the implementation of the algorithms in this research, CoxPH and RSF were implemented using the Python package “Scikit-learn (version 0.24.1)” and DeepSurv was implemented using the open-source Python package “Tensorflow-gpu (version 2.6.2)”.

### Model evaluation

The study evaluated the model’s performance using several metrics, including C-index, Brier score, integrated brier score (IBS), receiver operating characteristic (ROC) curves, and area under the curve (AUC) values.

The C-index is a commonly used metric for evaluating the accuracy of survival predictions^21^. It measures the concordance or correlation between the predicted survival risk and the actual observed survival time. A C-index of 0.5 indicates random predictions, while a value of 1.0 indicates perfect predictions. The Brier score assesses the mean squared difference between the observed patient statuses (event occurrence or censoring) and the predicted survival probabilities. It ranges from 0 to 1, with 0 indicating a perfect match between predictions and observations. In practice, models with Brier scores less than 0.25 are considered useful^22,23^. The IBS is a metric that evaluates the overall performance of a survival model across all available time points^24^. It takes into account the model’s sensitivity and specificity to time-dependent events, providing a comprehensive measure of predictive accuracy. Receiver Operating Characteristic (ROC) curves are frequently used to assess a model’s sensitivity and specificity at various discrimination thresholds. The ROC curve plots the true positive rate against the false positive rate. The Area Under the Curve (AUC) values, which range from 0 to 1, are computed to quantify the overall performance of the model. A higher AUC indicates better discrimination ability. This study calculated AUC values to assess the model's performance at different time points: 1, 3, and 5-year survival rates.

### Statistical analysis

In the clinical data, continuous variables are expressed as mean ± standard deviation (SD), while categorical variables are described using frequencies and percentages. Statistical tests such as chi-square tests and unpaired t-tests are used to compare variables between groups.

## Result

### Basic characteristics

This study analysed data from 2,322 medulloblastoma patients registered in the SEER database between 2000 and 2019. Table 1 presents the demographic features of the patients, with 869 cases (37.42%) being female and 1,453 cases (62.58%) being male. The racial distribution was as follows: 185 patients (7.97%) were Black, 1,939 (83.51%) were White, and 198 (8.53%) belonged to other races. Regarding the subtypes of medulloblastoma, 329 patients (14.17%) had desmoplastic/nodular medulloblastoma (DMB), 1,866 (80.36%) had medulloblastoma, not otherwise specified (MB, NOS), and 127 (5.47%) had large-cell/anaplastic medulloblastoma (LC). In terms of surgical interventions, 1,616 patients (69.60%) underwent total resection, 244 (10.51%) underwent subtotal resection, 343 (14.77%) underwent local excision or biopsy, and 119 (5.12%) did not undergo surgery. Of the patients, 1,849 (79.63%) received chemotherapy, 1,766 (76.06%) underwent radiation therapy, and 713 (30.71%) died. The cutoff values for age and tumor size were determined using X-tile analysis **(**Fig. 2**)**. Specifically, 324 patients (13.95%) were ≤ 3 years old, and 1,998 patients (86.05%) were older than 3 years. Regarding tumor size, 314 patients (13.52%) had tumors ≤ 3.4 cm, 1,269 patients (54.65%) had tumor size > 3.4 cm, and the tumor size was unknown for 739 patients (31.83%).

**Table 1:** Characteristic distribution of data into raining sets and test sets.

Variables	Overall N (%)	Train cohort N (%)	Test cohort N (%)	*P*
Patients	2,322	1625 (69.98)	697 (30.02)	
Age				0.73
≤ 3	324 (13.95)	229 (14.09)	95 (13.63)	
> 3	1,998 (86.05)	1,396 (85.91)	602 (86.37)	
Sex				0.12
Female	869 (37.42)	625 (38.46)	244 (35.01)	
Male	1,453 (62.58)	1,000 (61.54)	453 (64.99)	
Race				0.57
Black	185 (7.97)	135 (8.31)	50 (7.17)	
White	1,939 (83.51)	1,349 (83.02)	590 (84.65)	
Other	198 (8.53)	141 (8.68)	57 (8.18)	
Histopathology				0.24
DMB	329 (14.17)	229 (14.09)	100 (14.35)	
MB, NOS	1,866 (80.36)	1,302 (80.12)	564 (80.92)	
LC	127 (5.47)	94 (5.78)	33 (4.73)	
Size (cm)				0.17
≤ 3.4	314 (13.52)	211 (12.98)	103 (14.78)	
> 3.4	1,269 (54.65)	896 (55.14)	373 (53.52)	
Unknown	739 (31.83)	518 (31.88)	221 (31.71)	
Surgery				0.14
Total resection	1,616 (69.60)	1,122 (69.05)	494 (70.88)	
Subtotal resection	244 (10.51)	175 (10.77)	69 (9.90)	
Local excision/Biopsy	343 (14.77)	238 (14.65)	105 (15.06)	
No evidence	119 (5.12)	90 (5.54)	29 (4.16)	
Chemotherapy				0.25
Yes	1,849 (79.63)	1,274 (78.40)	575 (82.50)	
No evidence	473 (20.37)	351 (21.60)	122 (17.50)	
Radiotherapy				0.33
Yes	1,766 (76.06)	1,214 (74.71)	552 (79.20)	
No evidence	556 (24.94)	411 (25.29)	145 (20.80)	
Status				0.46
Death	713 (30.71)	514 (31.63)	199 (28.55)	
Alive	1,609 (69.29)	1,111 (68.37)	498 (71.45)	

The predictive model was generated by partitioning the complete dataset into two mutually exclusive subsets. 70% of the dataset was allocated for the training set, while the remaining 30% was used for the testing set. Model generation was performed on 1,625 randomly assigned patients from the training set, while the accuracy of the model was estimated using 697 randomly assigned patients from the testing set. No statistically significant differences in characteristics were found between the two groups (refer to Table 1). Additionally, survival outcomes showed no differences between the two groups (refer to Fig. S1).

### Cox proportional-hazard (CoxPH) model

**Figure 3:**
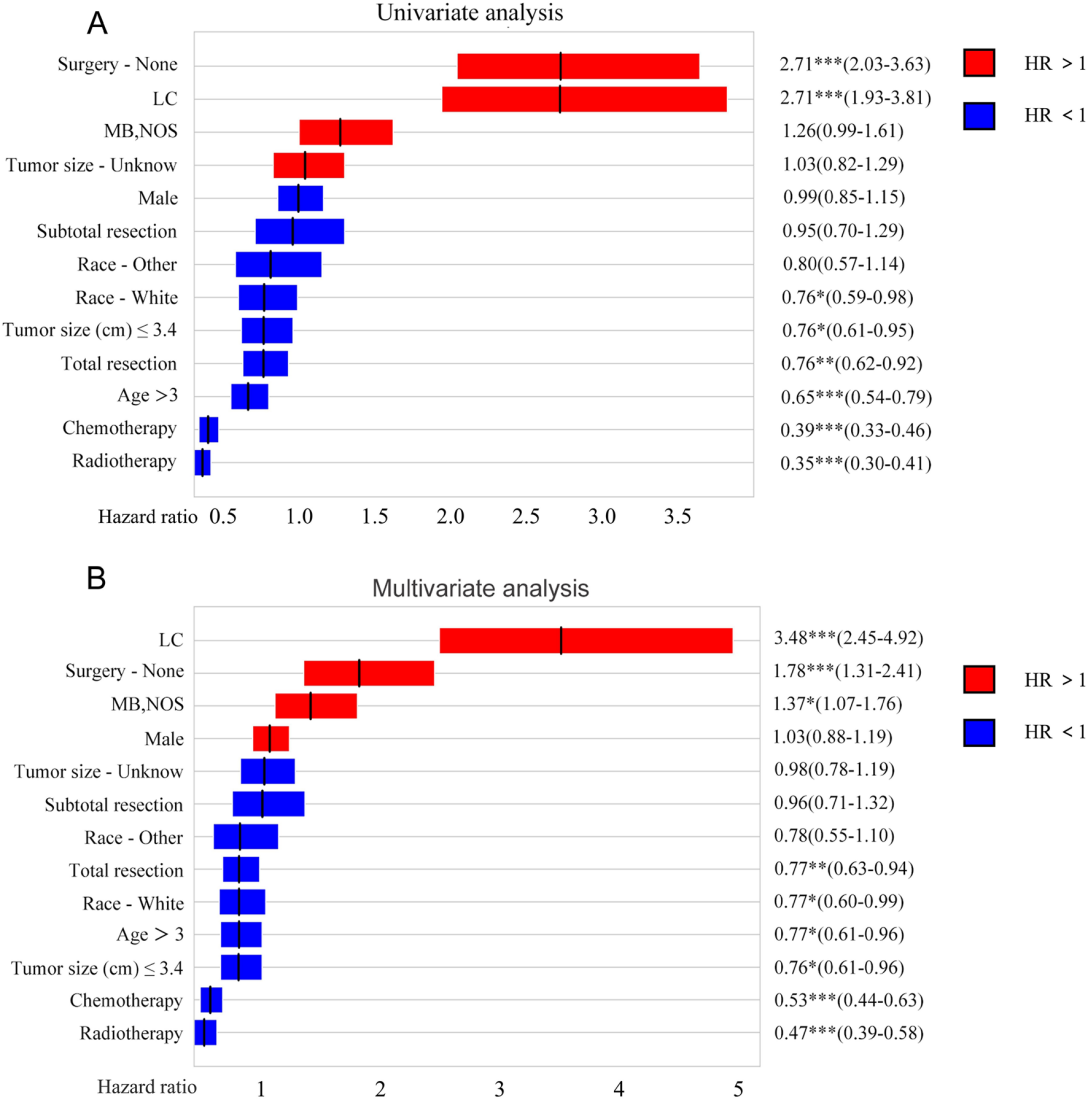
Univariate & Multivariable CoxPH analyses. Variables are sorted in descending order of hazard ratio (HR). Red represents a value above 1, while blue represents a value below 1. *p < 0.05, **p < 0.01, ***p < 0.001.

The CoxPH model was developed using the training set (refer to Fig. 3). Only variables that showed statistical significance in the univariate analysis were included in the multivariate analysis. The survival of medulloblastoma patients was significantly affected by non-surgical treatment, LC, white race, tumor size ≤ 3.4 cm, total resection, age > 3 years, chemotherapy, and radiotherapy. Furthermore, the survival of the patients was significantly associated with these features in the multivariate analysis. The collinearity analysis also revealed a high correlation between age and radiotherapy, as well as between chemotherapy and radiotherapy (refer to Fig. S2). Ultimately, we included seven features (age, race, tumor size, histological type, surgery, chemotherapy, and radiotherapy) in the model development.

### Random survival forests (RSF)

**Figure 4:**
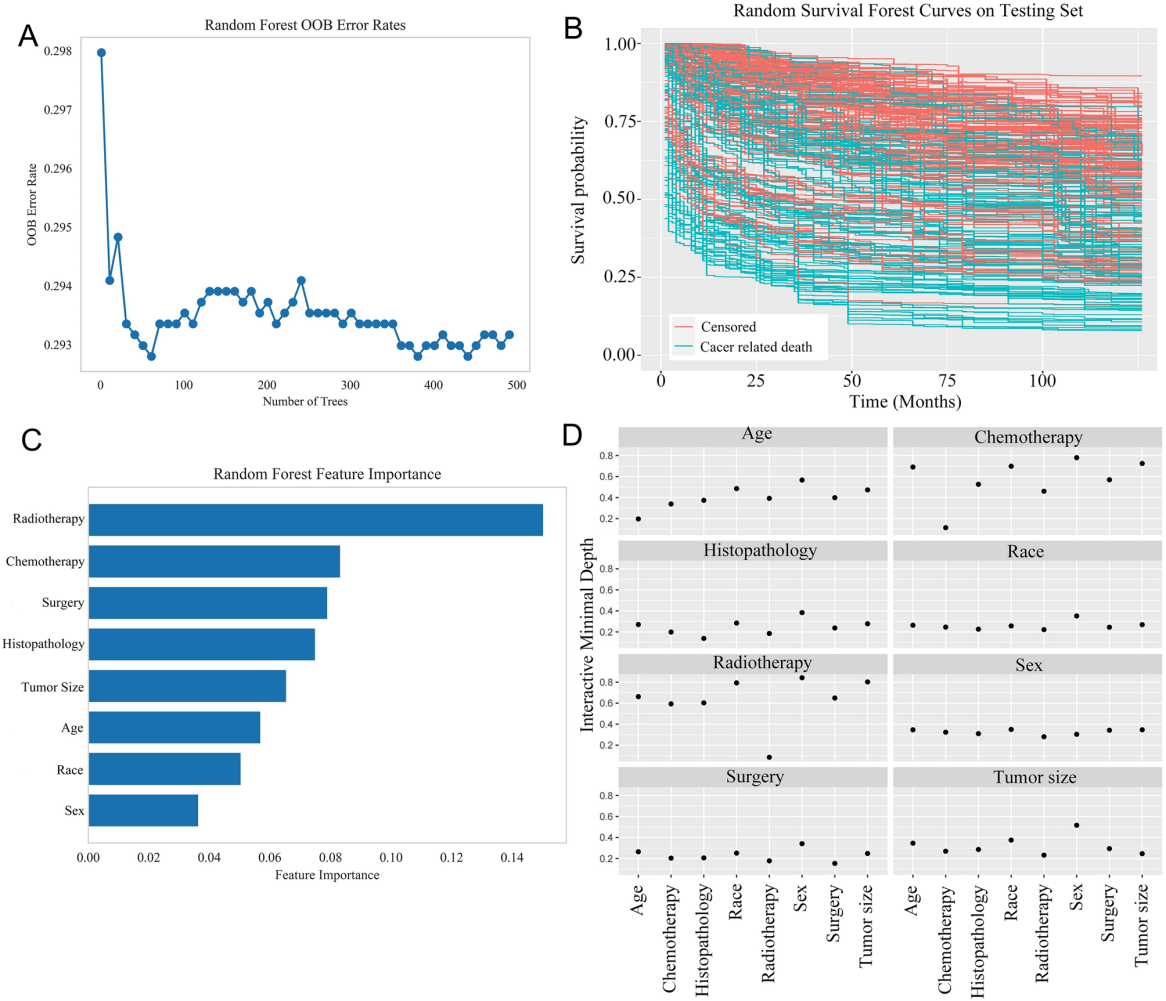
Random survival forest model. 8 features were used to construct the model: sex, age, radiotherapy, chemotherapy, histopathology, race, surgery, tumor size. (**A**) Out-of-bag (OOB) error rate. (**B**) Predicted survival curves generated for testing set. (**C**) Variable importance plot. Higher values of Variable Importance (VIMP) indicate the variable contributes more to predictive accuracy of the model. (**D**) Variable interaction plot. Lower values indicate a higher level of interactivity between the variables.

Prediction error was calculated using the out-of-bag (OOB) from the training set **(**Fig. 4A**)**. The predicted probability function for patient in the test cohort was plotted in Fig. 4B. Variable Importance (VIMP) is used to indicate the extent to which the sample characteristics contribute to the regression, as shown in Fig. 4C. A higher VIMP value indicates a greater influence or importance of that variable in accurately predicting the outcome^25^. The interaction between variables in the analyzed data is illustrated and displayed in Fig. 4D. If one variable's split in a decision tree affects or influences the split of another variable, it suggests an interaction between those variables^26,27^. The extent of interactions is assessed based on the minimum depth, which represents the distance from the root node to the node where the variable first splits. In this case, chemotherapy and radiotherapy were found to have the lowest minimum depth among the variables considered that were expected to be associated with other variables.

### DeepSurv

The hyperparameters of DeepSurv were tuned with reference to previous studies that grid search and fivefold cross-validation on the training dataset^18,28^. The model with the optimal set of hyperparameters achieved the accuracy of 91.06% and the corresponding R^2^ value of 0.6455. The best combination of the model hyperparameters included 2000 epochs, the Adam optimizer, binary cross-entropy loss, four layers (nodes: 32, 64, 128, 256), a dropout rate of 0.2, and a learning rate of 0.001. Furthermore, the performance of the model was evaluated with the testing set.

**Figure 5:**
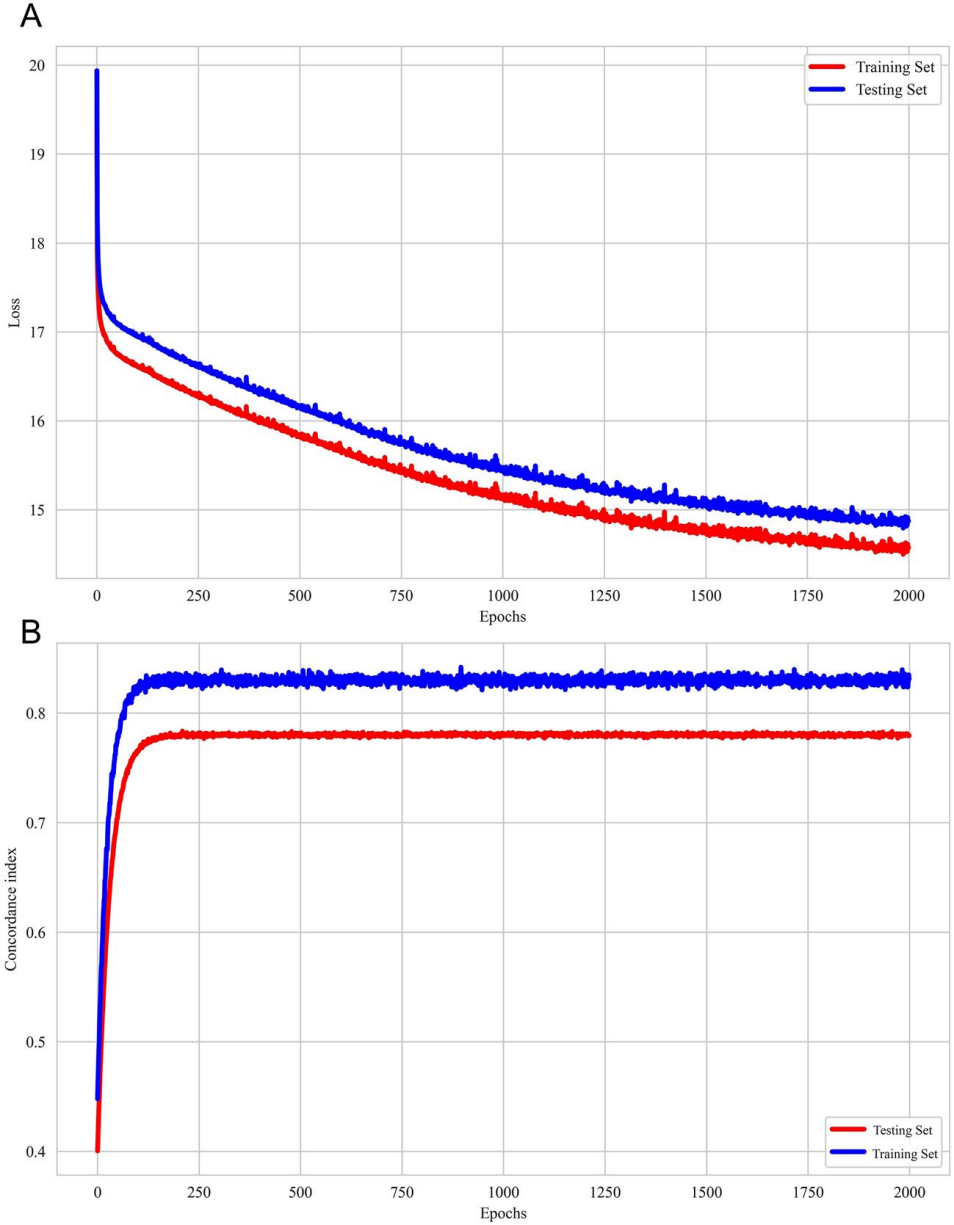
The training and testing history of DeepSurv. (**A**) A plot of loss on the training and testing sets. The error gradually decreases over each iteration during training. (**B**) A plot of the concordance index obtained by the model in the train and test sets as a function of the epochs. It is neither fitting the training data too well nor failing to capture important patterns in the data.

The loss function curve illustrates the relationship between the loss and the number of iterations, providing valuable information about the convergence and performance of the model^29^. In addition, the C-index is a commonly used metric for evaluating the performance of survival analysis models. If the C-index is only measured on the training set, the possibility of overfitting cannot be completely ruled out, as the model may over-fit the training data, leading to a decrease in generalization performance on test data^30^. In this study, the C-index was measured on two mutually exclusive data sets (training and test) and no overfitting phenomenon was observed. The learning process of DeepSurv, a survival prediction model based on deep learning, was visualized **(**Fig. 5**)**. The figure shown a good model fit, indicating that the model was effectively learning and capturing the underlying patterns in the data.

### Model comparisons

The predictive performance of the three models is shown in Table 2. In the test dataset, the DeepSurv and RSF model exhibited better discrimination abilities (the DeepSurv C-index: 0.763, RSF: 0.759) compared with the CoxPH model (the C-index: 0.757). And in the three models, DeepSurv had the highest C-index of 0.763. The C-index obtained from the train data set (DeepSurv: 0.751, RSF: 0.750, CoxPH: 0.748) differed only slightly with test set, indicating that the models did not exhibit overfitting. The IBS for the three models were as follows: DeepSurv (0.150), RSF (0.160), and CoxPH (0.166). Lower IBS values indicate better model performance.

**Table 2:** Performance of three survival models.

Models	C index		IBS	1-year ACU	3-year AUC	5-year AUC
	Train	Test				
CoxPH	0.748	0.757	0.166	0.736 (0.699–0.772)	0.712 (0.677–0.778)	0.704 (0.669–0.740)
RSF	0.750	0.759	0.160	0.757 (0.720–0.795)	0.738 (0.701–0.774)	0.734 (0.697–0.770)
DeepSurv	**0.751**	**0.763**	**0.150**	**0.793** (0.754–0.833)	**0.775** (0.736–0.814)	**0.767** (0.729–0.806)

Significant values are in bold.

**Figure 6:**
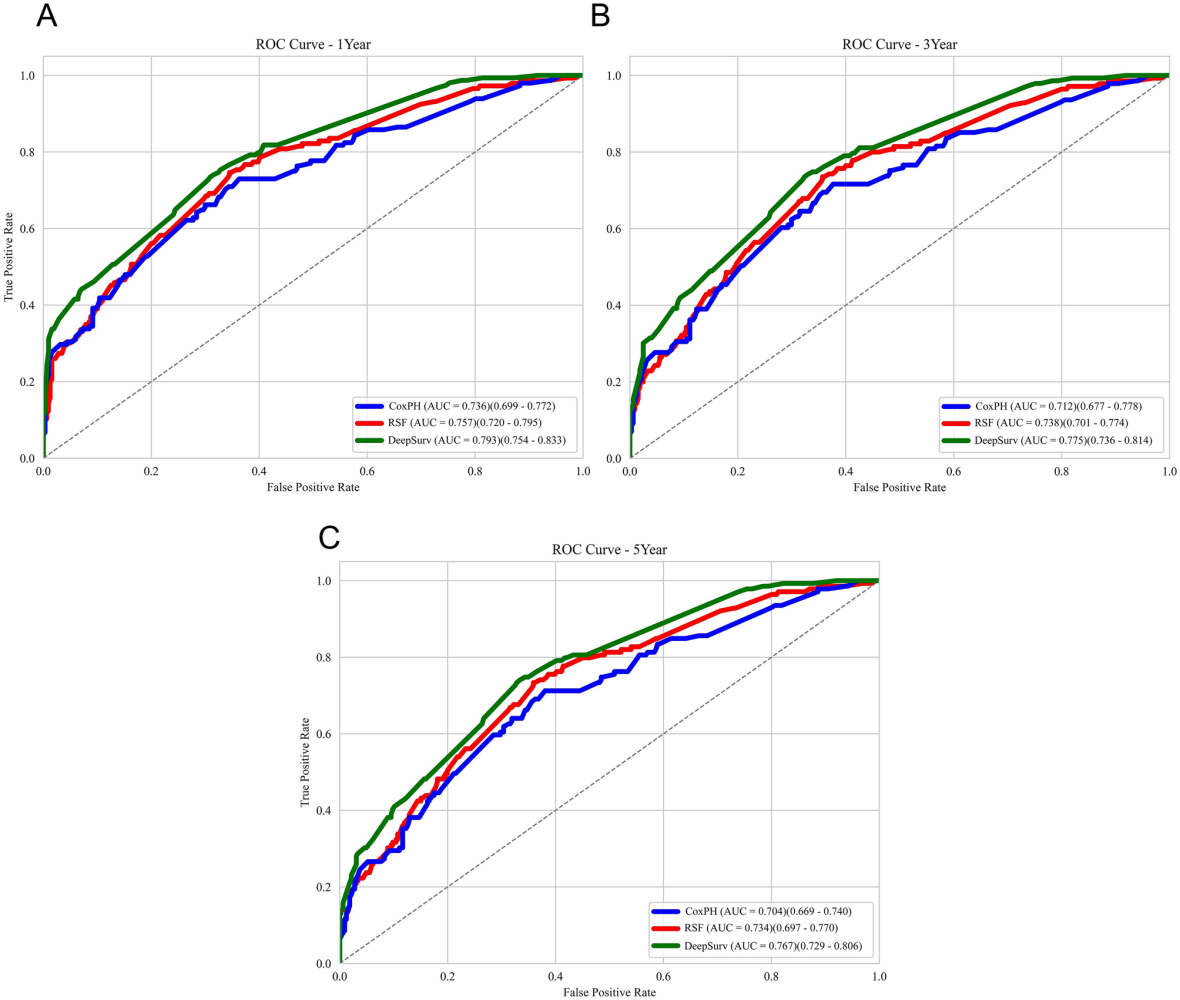
The receiver operating characteristic (ROC) curves for 1-year, 3-year, and 5-year survival predictions.

Furthermore, in terms of the Brier score (Fig. S3), DeepSurv outperformed the other two models, indicating its superior accuracy. The AUC for DeepSurv was also higher than the other models **(**Fig. 6**)**, that 1-year-AUC of DeepSurv: 0.793 (95% CI 0.754–0.833), RSF: 0.757 (95% CI 0.720–0.795), CoxPH: 0.736 (95% CI 0.699–0.772); 3-year-AUC of DeepSurv: 0.775 (95% CI 0.736–0.814), RSF: 0.738 (95% CI 0.701–0.774), CoxPH: 0.712 (95% CI 0.677–0.778); 5-year-AUC of DeepSurv: 0.767 (95% CI 0.729–0.806), RSF: 0.734 (95% CI 0.697–0.770), CoxPH: 0.704 (95% CI 0.669–0.740). These results demonstrate that DeepSurv outperforms both RSF and the classical CoxPH model in accurately predicting the prognosis of patients with medulloblastoma.

## Discussion

Medulloblastoma, a malignant brain tumor that mainly impacts children, continues to pose a substantial obstacle in the field of pediatric oncology. Precisely predicting the individual prognosis of patients is crucial for customizing treatment approaches and enhancing survival rates. Prior research has identified several prognostic factors that affect the survival duration of medulloblastoma patients, including age, extent of surgical removal, and the administration of radiotherapy or chemotherapy^7,31,32^. Moreover, as medical advancements progress, an increasing amount of imaging data^5^ and genetic data^33^ are being analyzed for survival analysis of medulloblastoma patients. However, classical survival analysis methods, such as the Cox proportional-hazards model, assume a linear relationship between variables, which may be limited in the face of multidimensional data. With the advancement of artificial intelligence, machine learning methods are being applied to clinical, imaging, and genetic data, allowing for the discovery of potential nonlinear relationships within the data^34–36^. Within machine learning, deep learning is a specific class of methods that utilizes multilayered neural networks to extract high-order features. Deep learning has gained increasing popularity in the field of cancer survival analysis, and has demonstrated excellent performance^37–39^. As far as we know, this approach has not been applied to medulloblastoma. Therefore, we applied a deep learning model (Deepsurv) to predict the overall survival (OS) of medulloblastoma patients and compared its performance to that of a machine learning model (RSF) and a classical model (CoxPH).

By extracting potentially significant features from the SEER database, this research developed multiple models to forecast the survival rates of individuals diagnosed with medulloblastoma. Initially, we utilized the X-tile tool to determine the optimal cutoff values for age and tumor size from a cohort of 2,322 medulloblastoma patients. We identified two high-risk factors, age ≤ 3 years old and tumor size > 3.4 cm, that significantly impact the survival duration of patients with medulloblastoma. Subsequently, we employed Cox proportional hazards regression to identify variables associated with the prognosis of medulloblastoma patients. Age, race, tumor size, histological type, surgery, chemotherapy, and radiotherapy were selected for inclusion in the modeling process (*p* < 0.05). We established RSF, DeepSurv and CoxPH models and evaluated their performance using metrics such as the C-index, IBS, and ROC curve. The study results demonstrated that the DeepSurv model outperformed both the CoxPH and RSF models, as indicated by its higher C-index in both the training and testing sets. Moreover, the DeepSurv model exhibited the lowest IBS and the largest AUC values when predicting 1-, 3-, and 5-year survival. These findings collectively suggest that the DeepSurv model is more accurate in predicting the survival of patients with medulloblastoma.

In previous studies, Guo et al.^7^ and Zhou et al.^5^ utilized Cox proportional hazard regression for survival analysis of medulloblastoma and developed a nomogram. Compared with their study, the C-index values obtained from the DeepSurv model were higher in both the training and the testing cohort, indicating its superior predictive accuracy of the prognosis of patients with medulloblastoma. This finding is consistent with the results reported in several previous studies focusing on cancer prognosis^40,41^. The main advantage of the DeepSurv model is its ability to handle both linear and non-linear predictive variables using a multi-layer neural network. It has a powerful ability to capture arbitrarily complex non-linear interactions in the data, allowing such models to discover correlations that are difficult for the human eye or traditional statistical techniques to detect.

Nevertheless, our study encountered several limitations. Firstly, the data collected from the SEER database for medulloblastoma patients contain some missing information that may affect survival outcomes, including important details such as molecular subgroups, specific radiotherapy doses, and chemotherapy regimens. Among other things, molecular diagnostics are critical for treatment and prognosis prediction of tumours, especially medulloblastoma. Nevertheless, the availability and completeness of these data depend on continuous improvements in data collection in the SEER database. Secondly, our model has yet to undergo external validation, and it is necessary to validate its performance on new data. Conducting further validations using independent datasets would enhance the reliability and generalizability of the findings. Another inherent limitation lies within the DeepSurv model itself. Due to its utilization of hidden layers in its architecture, the model operates as a black-box, making it challenging to fully comprehend the computations involved in the model construction process and its associated limitations. Future research should aim to address these concerns and explore the inner workings of the model to improve interpretability.

## Conclusions

This study employed Cox proportional hazards regression analysis to examine the prognostic factors influencing medulloblastoma patients’ outcomes, which include age, race, tumor size, histological type, surgery, chemotherapy, and radiotherapy. Subsequently, we developed a groundbreaking DeepSurv prediction model, which exhibited strong predictive capabilities in assessing the prognosis of patients diagnosed with medulloblastoma. This innovative DeepSurv model holds significant potential in accurately predicting the survival duration of medulloblastoma patients.

### Supplementary Information

Supplementary Figures.

## Data Availability

The datasets analyzed during the current study are available in the SEER database repository (https://seer.cancer.gov/).

## References

[1] Gajjar AJ, Robinson GW (2014). Medulloblastoma-translating discoveries from the bench to the bedside. Nat. Rev. Clin. Oncol..

[2] Ostrom QT, Cioffi G, Waite K, Kruchko C, Barnholtz-Sloan JS (2021). CBTRUS statistical report: Primary brain and other central nervous system tumors diagnosed in the United States in 2014–2018. Neuro. Oncol..

[3] Taylor MD (2012). Molecular subgroups of medulloblastoma: The current consensus. Acta Neuropathol..

[4] Ramaswamy V, Taylor MD (2017). Medulloblastoma: From myth to molecular. J. Clin. Oncol..

[5] Zhou L (2023). Automatic image segmentation and online survival prediction model of medulloblastoma based on machine learning. Eur. Radiol..

[6] Li X, Gong J (2023). Survival nomogram for medulloblastoma and multi-center external validation cohort. Front. Pharmacol..

[7] Guo C (2020). External validation of a nomogram and risk grouping system for predicting individual prognosis of patients with medulloblastoma. Front. Pharmacol..

[8] Baek ET (2021). Survival time prediction by integrating cox proportional hazards network and distribution function network. BMC Bioinform..

[9] Iasonos A, Schrag D, Raj GV, Panageas KS (2008). How to build and interpret a nomogram for cancer prognosis. J. Clin. Oncol..

[10] Schwalbe N, Wahl B (2020). Artificial intelligence and the future of global health. Lancet.

[11] Hamet P, Tremblay J (2017). Artificial intelligence in medicine. Metabolism.

[12] Hunter DJ, Holmes C (2023). Where medical statistics meets artificial intelligence. N. Engl. J. Med..

[13] Connor CW (2019). Artificial intelligence and machine learning in anesthesiology. Anesthesiology.

[14] Bhat M, Rabindranath M, Chara BS, Simonetto DA (2023). Artificial intelligence, machine learning, and deep learning in liver transplantation. J. Hepatol..

[15] Choi RY, Coyner AS, Kalpathy-Cramer J, Chiang MF, Campbell JP (2020). Introduction to machine learning, neural networks, and deep learning. Transl. Vis. Sci. Technol..

[16] Greener JG, Kandathil SM, Moffat L, Jones DT (2022). A guide to machine learning for biologists. Nat. Rev. Mol. Cell Biol..

[17] Jiang C (2023). Predicting the survival of patients with pancreatic neuroendocrine neoplasms using deep learning: A study based on surveillance, epidemiology, and end results database. Cancer Med..

[18] Katzman JL (2018). DeepSurv: Personalized treatment recommender system using a cox proportional hazards deep neural network. BMC Med. Res. Methodol..

[19] Hankey BF, Ries LA, Edwards BK (1999). The surveillance, epidemiology, and end results program: a national resource. Cancer Epidemiol. Biomark. Prev..

[20] Rahman SA (2021). Prediction of long-term survival after gastrectomy using random survival forests. Br. J. Surg..

[21] Alexiuk M, Tangri N (2024). Prediction models for earlier stages of chronic kidney disease. Curr. Opin. Nephrol. Hypertens.

[22] Jiang F (2024). Automated machine learning-based model for the prediction of pedicle screw loosening after degenerative lumbar fusion surgery. Biosci. Trends.

[23] Ding H, Yuan M, Yang Y, Gupta M, Xu XS (2024). Evaluating prognostic value of dynamics of circulating lactate dehydrogenase in colorectal cancer using modeling and machine learning. Clin. Pharmacol. Ther..

[24] Wang X (2023). Quantifying and interpreting the prediction accuracy of models for the time of a cardiovascular event-moving beyond c statistic: A review. JAMA Cardiol..

[25] Taylor JM (2011). Random survival forests. J. Thorac. Oncol..

[26] Gilhodes J (2017). Comparison of variable selection methods for high-dimensional survival data with competing events. Comput. Biol. Med..

[27] Kretowska M (2018). Tree-based models for survival data with competing risks. Comput. Methods Progr. Biomed..

[28] Adeoye J (2021). Deep learning predicts the malignant-transformation-free survival of oral potentially malignant disorders. Cancers.

[29] Du J, Zhou Y, Liu P, Vong CM, Wang T (2023). Parameter-free loss for class-imbalanced deep learning in image classification. IEEE Trans. Neural Netw. Learn. Syst..

[30] Serghiou S, Rough K (2023). Deep learning for epidemiologists: An introduction to neural networks. Am. J. Epidemiol..

[31] Dasgupta A (2019). Nomograms based on preoperative multiparametric magnetic resonance imaging for prediction of molecular subgrouping in medulloblastoma: Results from a radiogenomics study of 111 patients. Neuro. Oncol..

[32] Liu H, Sun P (2024). A nomogram model for predicting prognosis of patients with medulloblastoma. Turk. Neurosurg..

[33] Zhu S (2020). Identification of a twelve-gene signature and establishment of a prognostic nomogram predicting overall survival for medulloblastoma. Front. Genet..

[34] Erickson BJ, Korfiatis P, Akkus Z, Kline TL (2017). Machine learning for medical imaging. Radiographics.

[35] Eraslan G, Avsec Z, Gagneur J, Theis FJ (2019). Deep learning: New computational modelling techniques for genomics. Nat. Rev. Genet..

[36] Handelman GS (2018). eDoctor: Machine learning and the future of medicine. J. Intern. Med..

[37] She Y (2022). Deep learning for predicting major pathological response to neoadjuvant chemoimmunotherapy in non-small cell lung cancer: A multicentre study. Ebiomedicine.

[38] Tran KA (2021). Deep learning in cancer diagnosis, prognosis and treatment selection. Genome Med..

[39] Foersch S (2023). Multistain deep learning for prediction of prognosis and therapy response in colorectal cancer. Nat. Med..

[40] Huang B (2023). Deep learning for the prediction of the survival of midline diffuse glioma with an H3K27M alteration. Brain Sci..

[41] Zhang X (2023). Deep learning-based pathology image analysis predicts cancer progression risk in patients with oral leukoplakia. Cancer Med..

