# Supplementary material for: Deep learning models for predicting the survival of patients with medulloblastoma based on a surveillance, epidemiology, and end results analysis
Source: Sci Rep. 2024 Jun 24;14:14490. doi: 10.1038/s41598-024-65367-9 (PMC11196279; doi:10.1038/s41598-024-65367-9)
Supplement: Supplementary file 1 — Supplementary Figures. [file 41598_2024_65367_MOESM1_ESM.docx]

**Deep learning models for predicting the survival of patients with medulloblastoma based on a surveillance, epidemiology, and end results analysis**

Meng Sun^1^, Jikui Sun^1^*, Meng Li^1^*

^1^ Department of Neurosurgery, The First Affiliated Hospital of Shandong First Medical University, Jinan, Shandong 250014, China

* Corresponding author at: The First Affiliated Hospital of Shandong First Medical University, Jinan, Shandong 250014, China.

E-mail address: jikuisun2015@163.com (Jikui Sun), drlimeng@126.com (Meng Li).


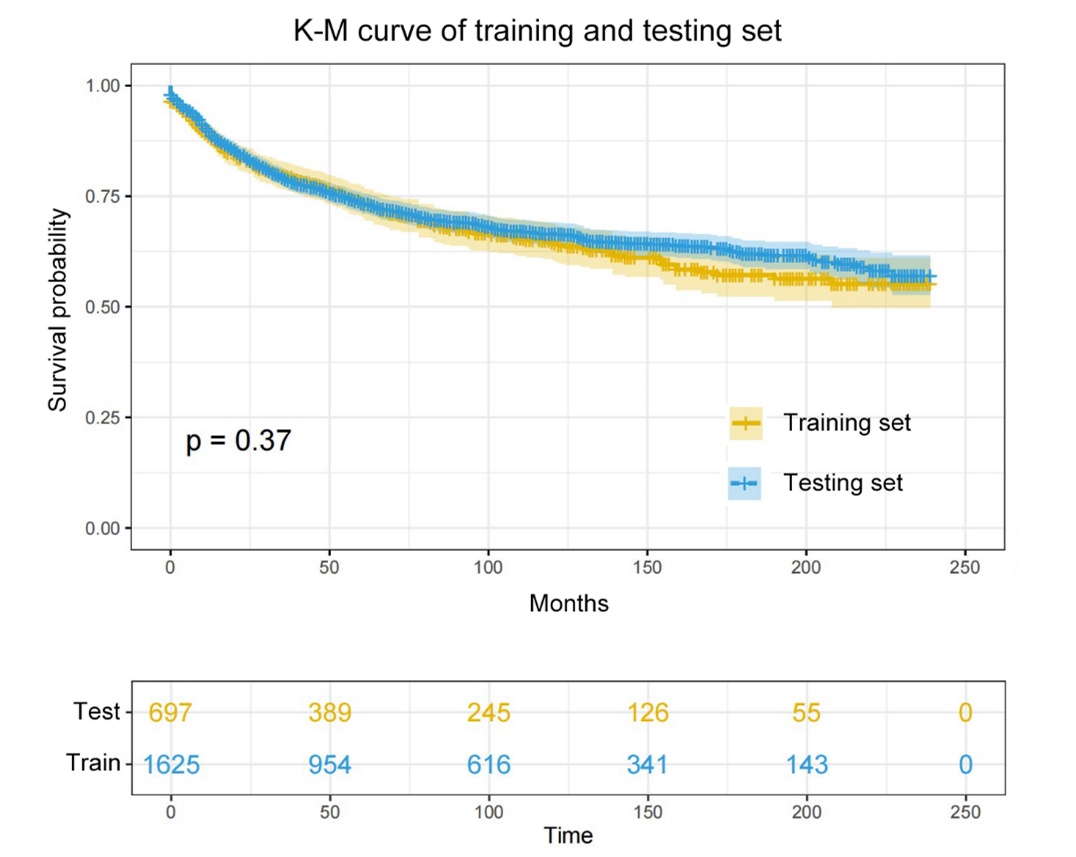


**Fig. S1** Kaplan-Meier curve of training and testing sets. There was no statistically significant difference between the survival of training and testing sets in log-rank test (*p* =0.37).


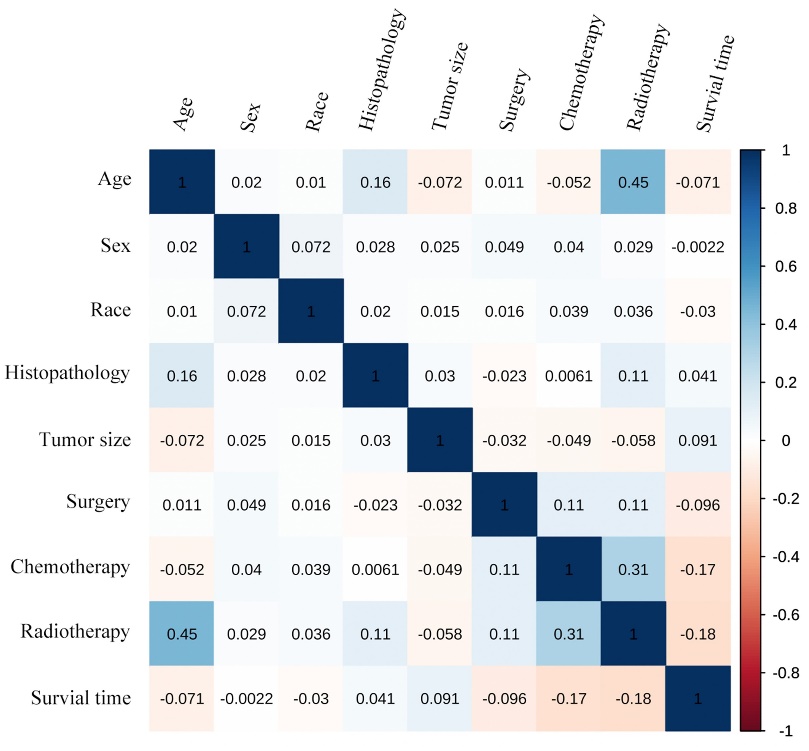


**Fig. S2** The correlation coefficients were calculated for each pair of variables in the dataset. These coefficients represent the strength and direction of the relationship between the variables and range from -1 to +1. The correlation values are displayed using color depth, with values closer to -1 or +1 indicating a stronger negative or positive correlation, respectively.


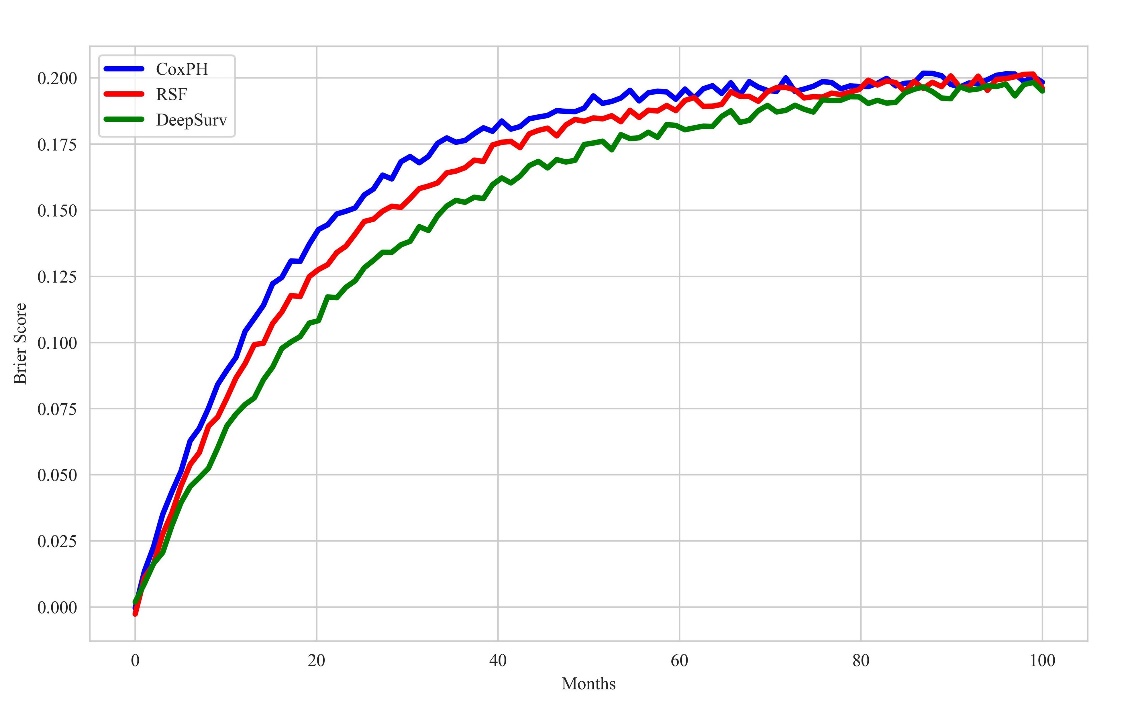


**Fig. S3** A plot of the Brier Score as a function of survival time. As a benchmark, a reliable model should aim for a Brier score below 0.25.
